# Problems Encountered in Fluctuating Flame Temperature Measurements by Thermocouple

**DOI:** 10.3390/s8127882

**Published:** 2008-12-04

**Authors:** Nadir Yilmaz, Walt Gill, A. Burl Donaldson, Ralph E. Lucero

**Affiliations:** 1 Department of Mechanical Engineering, New Mexico Institute of Mining and Technology, Socorro, New Mexico 87801, USA; 2 Sandia National Laboratories, Albuquerque, New Mexico 87185, USA; E-Mails: wgill@sandia.gov; bdonald@sandia.gov; 3 Department of Mechanical Engineering, New Mexico State University, Las Cruces, New Mexico 88003, USA; E-Mail: rallucer@nmsu.edu

**Keywords:** Fluctuating flames, Diffusion flames, Flame thermocouple measurement, Thermocouple transfer function

## Abstract

Some thermocouple experiments were carried out in order to obtain sensitivity of thermocouple readings to fluctuations in flames and to determine if the average thermocouple reading was representative of the local volume temperature for fluctuating flames. The thermocouples considered were an exposed junction thermocouple and a fully sheathed thermocouple with comparable time constants. Either the voltage signal or indicated temperature for each test was recorded at sampling rates between 300-4,096 Hz. The trace was then plotted with respect to time or sample number so that time variation in voltage or temperature could be visualized and the average indicated temperature could be determined. For experiments where high sampling rates were used, the signal was analyzed using Fast Fourier Transforms (FFT) to determine the frequencies present in the thermocouple signal. This provided a basic observable as to whether or not the probe was able to follow flame oscillations. To enhance oscillations, for some experiments, the flame was forced. An analysis based on thermocouple time constant, coupled with the transfer function for a sinusoidal input was tested against the experimental results.

## 1. Introduction

**Figure 1.**
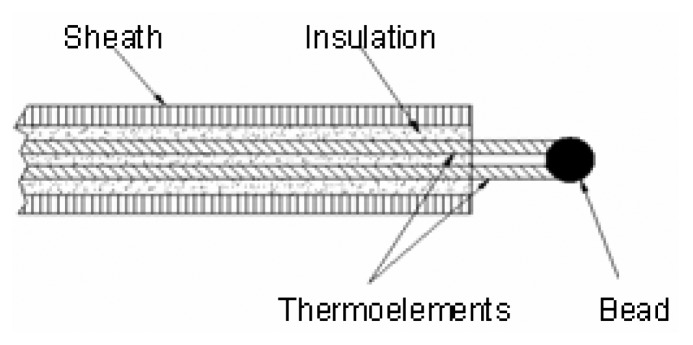
Cross-section of an exposed junction thermocouple probe.

**Figure 2.**
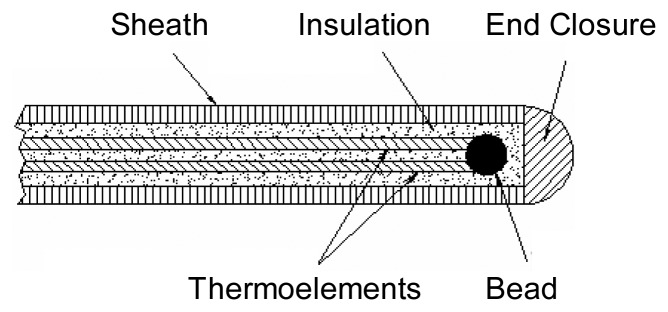
Cross-section of a sheathed thermocouple probe.

Thermocouples have been used extensively throughout industry to make temperature measurements in a variety of engineering situations. Such devices have been relied on extensively to measure the temperature of hot gases present inside laboratory and large scale outdoor flames. They are the most widely used thermal probes for flames [1]. Thermocouples are the temperature measuring devices of choice, because they have relatively fast response times if appropriately sized, can withstand high temperatures by appropriate material selection, are rugged, and are low cost. Thermocouples can be either bare junction. as shown in Figure 1, or with the junction enclosed by a metal sheath which provides electrical isolation from the environment, as shown in Figure 2. In the latter case, thermal energy must conduct through the metal sheath and insulation before a change in thermoelectric voltage at the circuit can develop. Figure 1 and Figure 2 are not to scale, and in fact, the size of the bead for the bare junction is larger than the diameter of the sheath for the enclosed configuration in this study. However, in compensation for the larger thermal mass, there is no thermal insulation associated with the bare junction to insulate the junction from the environment, as is the case with the sheathed thermocouple.

Interpretation of thermocouple readings in a flame must be made with the assistance of an appropriate energy balance model because there are various heat exchange mechanisms at play, depending on type and size of flame, and local events, including flow fluctuations and variable radiant path lengths between the probe and surroundings both inside and outside the flame. With an appropriate heat balance model for a particular flame, the thermocouple readings may be corrected for net energy transfer including radiation with the environment that the thermocouple “sees,” and convection heat transfer from hot combustion gases. Such corrections have been addressed by various researchers in flame temperature measurements [2-3]. More specifically, in clean premixed laboratory burner scale flames, the indicated temperature measurement may be as much as 800 °C or more below the combustion gas temperature (calculated from adiabatic equilibrium and confirmed experimentally by CARS [4]). This is due to the thermocouple energy balance that includes input from combustion gas convection and surface heat loss to the surroundings outside the flame by radiation from the thermocouple; this factor becoming very large when the thermocouple surface reaches temperatures of 1,200-1,500 K. In clean laboratory flames, the participation by gas band emission or absorption from carbon dioxide and water vapor are negligible and soot is not present in abundance. In small laboratory diffusion flames where soot is present, there will be a component of radiation which depends on both the soot volume fraction and on radiation path lengths. In very large flames, such as pool fires, the soot volume fraction can be large, and the path length through the flame to the surroundings can be large so that the thermocouple is not in communication with surfaces outside the flame. Hence, utilization of thermocouples in flames requires a rather sophisticated method for performing an appropriate energy balance which accounts for the different environments that the thermocouple “sees.” It is typical to use a computer program or fire code for this purpose. In this manner, the flow field, the local convection coefficient to the thermocouple, an estimate of the soot volume fraction, the path length for radiation, all can be taken into account so that the thermocouple reading can be interpreted. A presentation of such an effort is discussed extensively in [4]. Although there is still some unresolved uncertainty, this study found that such a fire code can account for more than 90% of the 800 °C discrepancy noted above.

Fire codes predict average values in a particular region of the flame so do not consider the influence of local fluctuations in temperature of both combustion gases, and soot radiation in the vicinity of a thermocouple. It is a recognized fact that both small scale and large scale fluctuations will occur in diffusion flames where combustion air is inducted in a seemingly chaotic fashion in synchronization with oscillation in the flame envelope. These oscillations arise due to buoyancy, acceleration and diffusion effects. A significant component of the fluctuation is periodic, although not all. Reynolds, Froude and Strouhal numbers are important non-dimensional parameters to analyze the effect of oscillations on flames [5-7]. Depending on the thermocouple time constant and the frequency of oscillation, the thermocouple probe will time-average fast fluctuations but slow fluctuations can be found in the trace. Large rugged sheathed thermocouples, 1/4″ (6.35 mm) in diameter for example, have poor time resolution and thus are not very sensitive to short time and small scale fluctuations. Small thermocouples on the order of 10 μm in diameter offer high precision and resolution with minimal disturbance of the flame. These are particularly useful when well-resolved temporal information is desired. It has been shown that decreasing the size of the junction yields a much faster response time and also increases the spatial resolution. However, because the convection coefficient increases inversely with diameter, many metals will not survive the adiabatic flame temperature of premixed fuel/air flames when the thermocouple diameter becomes very small. Hence, the thermocouple should be sized no smaller than is appropriate to the resolution desired. While fire codes are useful in interpretation of a thermocouple reading, the fire code will not resolve small scale fluctuations, so only the average values can be compared.

For much testing involving large outdoor fires, ungrounded, fully sheathed 62 mil OD (1.575 mm), type K thermocouples are used. The large dimension provided for ruggedness in handling, and unless time varying events on a scale of milliseconds are expected, then response time is generally not a concern. The ungrounded junction configuration is frequently selected because large fires create a high level of random electrical noise. This noise is picked up on grounded or exposed junction thermocouples, and leads to a low signal to noise ratio, thus reducing sensitivity to the desired signal. Because of electrical isolation of the ungrounded thermocouples, its output is less influenced by electrical noise emanating from the fire. Because of these factors, local flame oscillations on a short time scale generally go undetected. Longer oscillations, which may be due to variable wind speed and direction or puffing of the flame envelope, are quite apparent in the indicated readings.

As a scoping study, two styles of small thermocouple probes were placed inside a methane diffusion flame to determine if small, sheathed and unsheathed thermocouples were sufficiently fast in response to sense flame oscillations at rates consistent with what might be expected in a diffusion flame. Additionally, can an off-set of average temperature be noted as a consequence of forced oscillations which might stimulate near-thermocouple influences at one location in a large fire as a consequence of oscillations in neighboring regions?

## 2. Thermocouple Model

Time constants are typically used to indicate how fast the thermocouple will respond to a step change in its environmental temperature and this value is commonly known for various thermocouples when exposed to a given environment, or can be easily measured. A generally accepted definition for time constant is the time required for the instrument to change reading by a fraction equal to (1-1/e), i.e., 63.2%, of the change in the environmental temperature. The time constant is based on a first order, linear differential equation which presumes that heat input is by gas convection. Provided that the temperature difference between the environment and the sensor is not great, any radiation contribution to heat exchange can be linearized and included with the convection coefficient. So, the value for the time constant is not only a function of thermocouple size and type, but is also a function of the environment to which the thermocouple is exposed. For both the sheathed 10 mil thermocouple and the bare thermocouple used in the present experiments, the time constant was found from the Omega Temperature Handbook [8] to be on order of 0.5 seconds in a particular flowing hot air stream. While there will be some physical differences between combustion gases and air, from [4], the time constant for a 62 mil, sheathed thermocouple inserted into clean flame gases was 5 seconds; the corresponding value from [8] was 6 seconds.

The transfer function relates the output of the thermocouple to the input which drives it; more specifically, the Laplace transfer function is defined as the ratio of the Laplace transform of the output quantity to the Laplace transform of the input quantity when all initial conditions are zero. Doebelin [9] provides an excellent analysis of thermocouples, which he describes as first-order instruments. Doebelin also presents the response of thermocouple output to a sinusoidal variation in the input function. Although the natural and forced oscillations for flames are not purely sinusoidal, Doebelin's analysis provides a convenient way to consider the thermocouple response to periodic behavior. From the analysis presented by Doebelin, the sensor frequency does not change from the input signal, but is shifted by a phase angle of tan^-1^(ωτ) where ω is the forcing function (flame) frequency, and τ is the thermocouple time constant. The ratio of amplitude of the response to the amplitude of the stimulus is given by the reciprocal square root of (ω^2^τ^2^+1).

## 3. Experimental Procedure

The basic setup used during the acquisition of thermocouple data involved attaching thermocouple probes to a National Instruments analog/digital low voltage data acquisition board. The board was connected to a high resolution National Instruments data acquisition card (PCI 6025-E) with built-in analog-to-digital converter. The signal was acquired, saved, and analyzed using National Instruments LabView® software. The setup had the capability of visualizing the signal in real time and performing all the post- processing at the same time the signal was being acquired. The data collection system was equipped with an internal reference to avoid the need to set up and maintain an ice bath reference junction.

The thermocouple tested was placed approximately 3/4″ (19.05 mm) above a slot burner orifice, the opening dimensions were 1/32″ (0.794 mm) wide by 2″ (50.8 mm) long. The slot burner was fastened securely inside an enclosure which was used to prevent any unwanted room air movement which might cause the flame structure to be influenced by external sources. The enclosure was ported in the bottom and lower sides with ¼″ holes to supply combustion air during the experiment.

The indicated flame temperature was measured during tests which need a flow rate for which strong oscillations, both natural and forced, existed in the flame. Tests involved two different types of probes, as well as forced and unforced fuel flow conditions. By forcing or oscillating the fuel flow, the flame could be made to oscillate at off-natural frequency or to amplify natural frequency.

To determine whether or not the thermocouple probes were capable of sensing specific frequencies, the flame was forced to oscillate at known frequencies between 5-12 Hz, and the resulting voltage or temperature signal was recorded. Forced oscillation was accomplished by perturbing the supply of methane gas where it passed through a cavity formed by a 12″ (304.8 mm) subwoofer and a plastic five-gallon (0.0189 cubic meter) bucket. The subwoofer, because of its low frequency response, was used to drive the perturbations. The driving signal was produced by a Fluke arbitrary waveform generator and amplified by a 250W Optimus power amplifier, which also delivered the signal to the subwoofer. The power amplifier allowed the flame to be driven up to its maximum amplitude per the flow conditions, without blow-off. The amplitude of the pulsations was carefully adjusted, using coarse and fine adjust variable resistors, or potentiometers.

**Figure 3.**
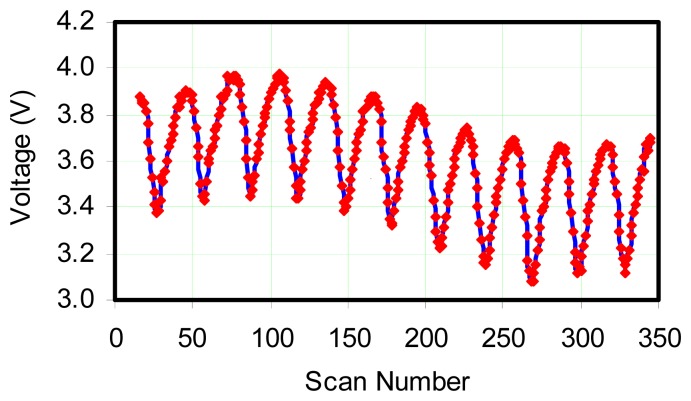
Photocell signal. Acoustic forcing was applied to flame at 10 Hz and was data shown is the photocell output. The above signal was used to verify that the flame was indeed oscillating at the forced frequency. Flow rate was 1.7 SCFM (2.888 m^3^/hour).

When not driven at the forcing amplitude, the flame oscillated at a specific natural frequency which is related to burner design, gas flow rate, and other factors. In order to confirm that the flame was being driven at a forced frequency from the waveform generator, the flame frequency was measured using a cadmium sulfide photocell. For this confirmation, the waveform generator was set to 10 Hz and the flame was forced to oscillate at this nearly natural frequency. The voltage across the photocell was sampled at a rate of 300 Hz, and then peak-to-peak measurements were made. Figure 3 shows the photocell output voltage when exposed to the oscillating flame. By making a simple peak-to-peak measurement, the frequency was measured and matched the value set by the waveform generator--10 Hz. It also appears that there is a lower frequency present in this trace. For later purposes, the amplitude variation in output signal shown in Figure 3 is roughly 14%. Since this sensor is responsive to light, it can be conjectured that the radiation environment (solid soot radiation) from the flame was oscillating at this frequency. Because soot particles are small, it is the consensus in the combustion community, that gas temperature and soot temperature track together, i.e., both undergo the same fluctuations in amplitude. This comment is admittedly an oversimplification because it does not include optical thickness, communication with environment, the wide angle “seen” by the photocell outside the flame and other complicating factors. Because of these moderating factors, it can be hypothesized that the true amplitude of temperature oscillation in the flame is substantially higher than 14%. At this point, the frequency of flame oscillation was varied and temperature measurements were made at various flow rates of fuel to provide information about how flame oscillations affect the thermocouple signal.

## 4. Results and Discussion

In the following sections, results from: 1) a bare junction thermocouple in a fluctuating flame environment, 2) an ungrounded, sheathed junction thermocouple, in a fluctuating flame environment, and 3) measurement of off-set average temperature as a function of oscillating frequency, will be presented and discussed. Type K thermocouple probes were used exclusively in this study because of their common use throughout industry, compensation with available software and excellent operating ranges.

### 4.1. Type K Thermocouple with Bare Junction

This test involved the use of a Watlow G281535 62 mil (1.575 mm) diameter Inconel sheathed Type K thermocouple but in which the bead or junction was exposed, as depicted in Figure 1. According to ASTM standards [10], the thermoelectric pair wire size will be 19% of the OD of the sheath, i.e., 12 mils (0.3 mm). However, the thermocouple junction will be approximately 3 × this dimension, i.e., 36 mils, or 0.9 mm. For this test, temperature data was sampled and acquired at a rate of 300 Hz. The fuel flow rate was 1.3 scfm (2.2087 m^3^/min) and was not pulsed.

**Figure 4.**
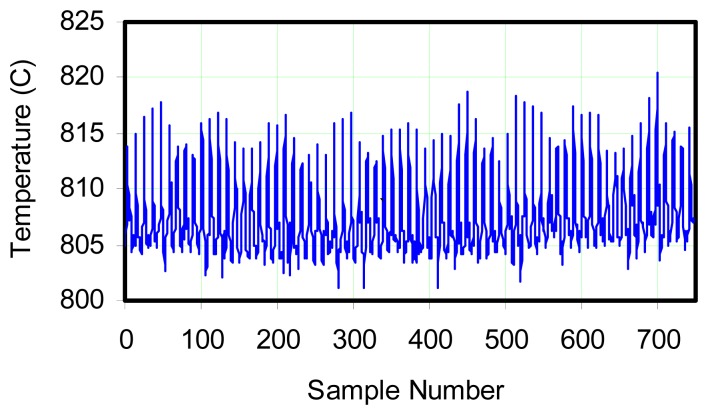
Temperature plot for exposed junction, Type K thermocouple with flow rate 1.3 SCFM (2.2087 m^3^/min), fuel supply not pulsed and sample rate 300 Hz. Average temperature is 807 °C.

As shown in Figure 4, temperature fluctuations obviously exist. It appeared that the temperature signal fluctuated at a more rapid rate than that of a naturally oscillating flame. It was known from other testing that the flame naturally oscillated at approximately 9 Hz. A simple peak-to-peak measurement of the data picked up a strong temperature fluctuation at around 27-29 Hz, three times the natural flame oscillation frequency. This suggests that vortex formation, which may be responsible for fluctuations in the signal, may occur at three times the expected natural frequency. To the eye, there appears to be another oscillation at around 3.5 Hz, but no oscillation is apparent at 9 Hz. However, a high level of noise can also be noted in this figure, which obviously confounded the extraction of a clear signal, and points up the deficiencies in using a bare thermocouple junction.

**Figure 5.**
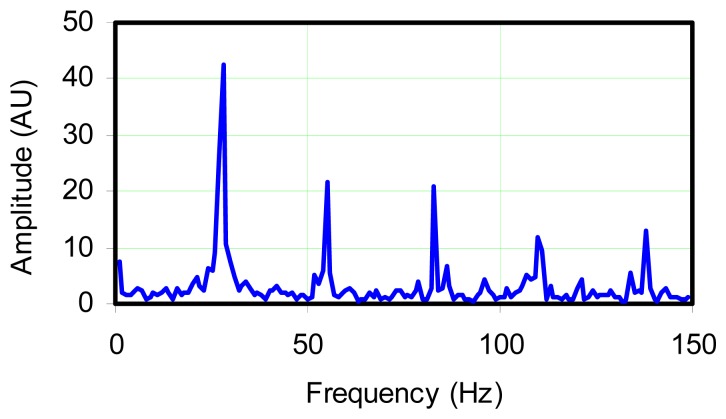
FFT analysis of data shown in Figure 4.

To further investigate the signal, a spectral graph was generated, using the FFT algorithm. The spectral graph in Figure 5 indicated that several frequencies existed in the trace. The strongest peak occurred at 29 Hz and because of the strength of the 29 Hz frequency, FFT analysis did not indicate significant oscillations at either 3.5 Hz, or 9 Hz, and the strength of oscillation at these frequencies is comparable to apparent random noise in the signal. The graph shows that the rest of the peaks occurred at higher frequencies which appear to be harmonics.

At this point, it is useful to consider the amplitude of the response recorded by the thermocouple. If figure 3 is presumed to correspond to the input to the thermocouple, a 14% variation has been noted (from the mean) for a 10 Hz oscillation frequency. Noting this approximately corresponds to the natural frequency of 9 Hz for the flame, then the amplitude of oscillation indicated by a thermocouple with a 0.5 sec time constant should be: 14%/[(10×0.5)2 +1] ∼ 0.5%. The oscillation shown in Figure 4 is ∼ 1.2% from mean. This reinforces the hypothesis that the oscillation amplitude sensed by the photocell was moderated by a substantial influence of the non-oscillating component in its environment.

### 4.2. Sheathed Type K Thermocouple

This test was conducted using a 10 mil (0.254 mm) OD stainless steel sheathed Type K ungrounded thermocouple but with 5 Hz forced oscillations produced by using a signal generator and subwoofer in the fuel supply. The thermoelectric signal was recorded and processed in the same fashion as in Test 1, except without reference compensation.

**Figure 6.**
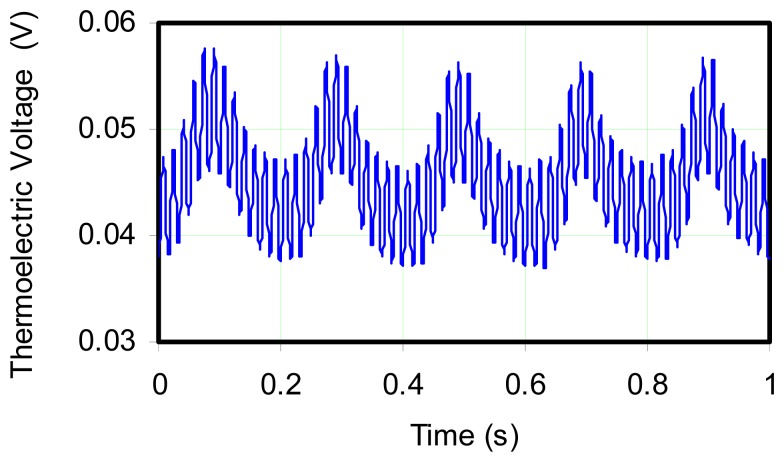
Unfiltered data for 10 mil sheathed, Type K thermocouple signal. Fuel subjected to 5 Hz pulsations; sample rate 1000.

**Figure 7.**
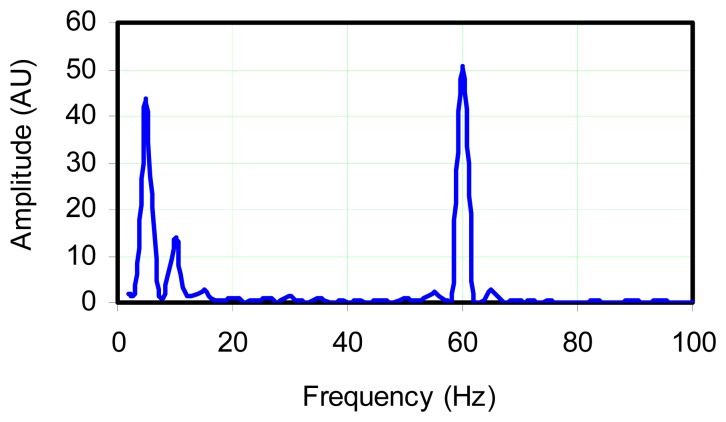
FFT analyzed Type K thermocouple signal for data set shown in Figure 6. Fuel supply subjected to 5 Hz pulsations; sample rate 1,000 Hz.

Figure 6 shows the unfiltered raw data, not converted to temperature in order to simply illustrate the time series showing superposition of fast and slow oscillations. A sampling rate of 1,000 Hz was used in the data acquisition. From the figure, it was immediately evident that the probe was able to detect the forced 5 Hz oscillations in temperature as well as other frequencies. Figure 7 shows a FFT of this signal which demonstrates the probe's ability to sense the 5 Hz and a weaker 10 Hz oscillation, as well as 60 Hz noise which is presumably caused by the electrical circuit in the lab. The magnitude of the 5 Hz and 60 Hz frequencies of approximately 4 mV, indicates the relative strength of the fluctuations.

The 5 Hz frequency seen in the FFT corresponded to the frequency at which the flame was being driven. The 10 Hz frequency was the natural frequency of the oscillating diffusion flame which is weakly apparent but was dominated by the forcing frequency. It was not possible to completely eliminate the natural frequency without extinguishing the flame. Driving the flame at the maximum possible amplitude without blowing it out is a difficult task. A simple solution which offered acceptable results, was fine-tuning the voltage across the subwoofer driver which was achieved through the use of fine and coarse adjustable potentiometers.

In comparison of the oscillation amplitude shown in Figure 6 to that which could be expected by applying the transfer function, the oscillation amplitude is found to be too high. While part of the problem may be that this signal was not referenced, another explanation is that the average temperature is substantially depressed due to the forcing. This depression will be examined in more detail in the following section.

### 4.3. Influence of Flame Oscillations on Average Thermocouple Temperature

**Figure 8.**
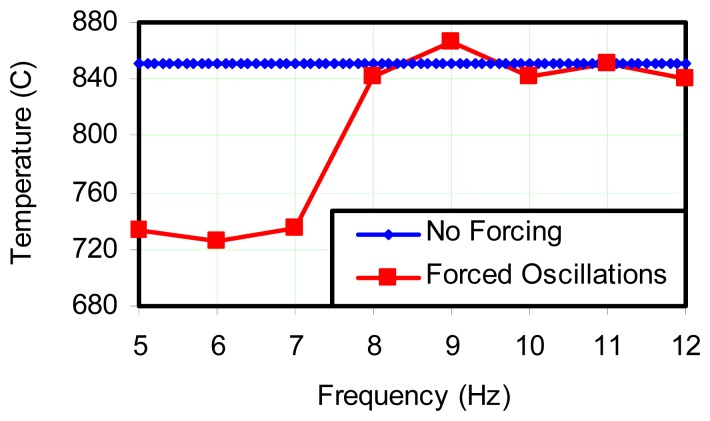
Indicated thermocouple temperatures for forced and unforced flame oscillations for bare junction Type K thermocouple for various frequencies. The straight line indicates average temperature while no forcing is present.

Large diffusion flame fires are highly inhomogeneous. Even above the fuel rich region there is significant turbulence, so that if a sensor is fixed in location, its output will be influenced by the environment in the local region. This can be due to radiant exchange which is a function of a “path length”, so that the thermocouple may see hotter regions in the flame as heat release occurs as a consequence of turbulent mixing. Or the thermocouple may see the external surroundings if wind or other perturbations reduce the path length between the thermocouple and the plume boundaries. In order to determine if there can be an offset in indicated average temperature sensed by a thermocouple in an oscillating flame, an experiment was conducted in which a maximum temperature zone was found above a slot burner while oscillating at its natural frequency of 9 Hz. With the thermocouple location maintained, the flame was forced to oscillate at 5 Hz, and the output was recorded continuously as forcing frequency gradually increased. Figure 8 shows the result which indicates rather conclusively that the oscillation frequency has a significant influence on the thermocouple output. But the effect diminishes as the oscillation frequency approaches the natural frequency.

**Figure 9.**
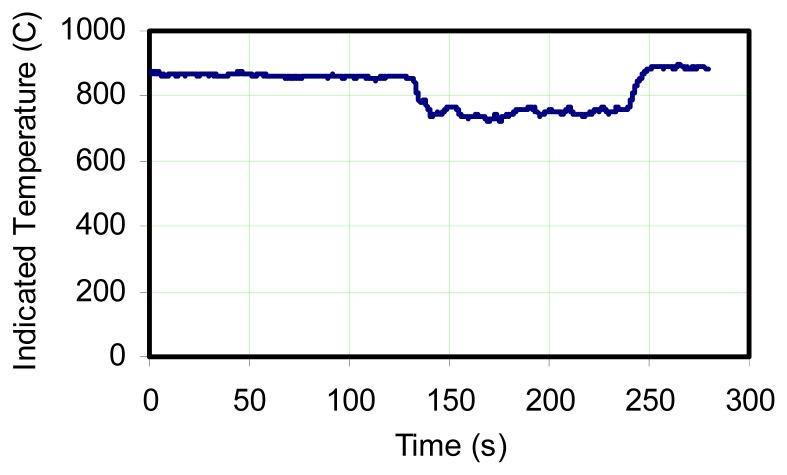
Thermocouple reading illustrating depression during forced oscillations of 5 Hz. Higher readings correspond to natural oscillation.

While it is not within the scope of this study to analyze the causes for the indicated temperature to drop, never-the-less, some speculation can be made. That is, the natural flame oscillation represents a natural coupling and optimization, which includes global reaction kinetics, air induction, and the production of soot. So the interdependent processes may couple in a manner which drives the most intense heat release at a specific location under natural conditions. This will enhance the buoyancy of the combustion gases and provide maximum acceleration of air to enter the reaction from outside the flame. Figure 9 is a similar plot where the oscillation frequency was varied between a forcing frequency of 5 Hz, and the natural frequency. This plot indicates that order of 100 °C+ differences can be experienced when the flame is not allowed to oscillate at its natural frequency.

There were several factors which may contribute to such a large change in average temperature. The first was that the thermocouple tip was sometimes in and out of the flame. This was mainly due to the fact that a large change in the shape of the flame envelope occurred during forced oscillation and, as a result, the thermocouple probe was sometimes outside the luminous region. Therefore, the probe was only partially exposed to the hot gases of the flame during the cycle. As a result, the probe's ability to sense ambient conditions was increased and in the case of a thin flame where the thermocouple can see outside the flame, the average temperature decreases. Conversely, if the flame structure is such that a thermocouple is in a location where it can see a higher temperature environment, its signal could be shifted upwards. Similar arguments could be made for large flames in which hot spots arise and diminish in the region of a thermocouple. If these hot spots occur randomly, then the average thermocouple reading should not see an off-set. However, large flames exhibit organization at several levels, and so are not exclusively random in behavior. Even with the 100 °C+ off-set noted in this experiment, this is not sufficiently high to rationalize the observation that the amplitude of oscillation in the sheathed thermocouple exceeded that which could be expected from the transfer function.

## 5. Conclusions

The primary motivation for this work was to examine the assertion that the average temperature which a thermocouple senses will not be the same time average that is predicted by the fire codes due to the nonlinear nature of radiation between the thermocouple and surroundings and between the thermocouple and soot which is produced in a diffusion flame. In other words, is the average reading shifted above the arithmetic average of the oscillation, and perhaps this shifting should be taking into account when attempting to validate the fire codes against experimental data? In order to try and evaluate this effect, an attempt was made to enhance the oscillation by forcing the flame and see if there was an upward shift in average thermocouple reading. However, the forced oscillation had the effect of changing the physical structure of the flame so that the maximum heat release no longer occurred at the same fixed location as a naturally oscillating flame, and hence, the effect showed a reduction in average temperature rather than an enhancement. So the findings reported here do not provide a basis for the examination of the effect in question and a different approach will need to be found.

A secondary objective of these experiments was to quantify the ability of a thermocouple placed inside an oscillating flame to provide useful data for characterizing the flame in terms of frequency response and magnitude of measurement. From the results, it was clearly evident that commonly available probe sizes and types were able to detect oscillations in flames at frequencies on order of natural flame oscillations. However, it is unlikely that oscillations at an order of magnitude higher frequency could be resolved with this size thermocouple unless the amplitude of the signal-to-noise ratio can be enhanced by appropriate filtering. By using the ungrounded and sheathed configuration, random noise is minimized, as compared to the bare junction thermocouple with a comparable time constant. These results also indicate that flame oscillations can influence the average thermocouple reading by changing the environment surrounding the thermocouple. The experiments reported here illustrate the complexities in attempting to measure temperature in flames using thermocouples. In retrospect, radiation analysis and scale of the flame should have been included in the study. A simple experiment would be to sample an oscillating flame simultaneously by both photocell and thermocouple, and with both signals on the same time base, it should be possible to determine phase shift. In this manner, a “dynamic” time constant could be obtained and compared to the traditional time constant for simple thermocouples. For sheathed thermocouples, the issue may be much more complex and require consideration of not only the thermal mass of the sheath and junction, but also the internal insulation. This comment is based on the observed amplitude of oscillation which was significantly higher than would be expected from the transfer function analysis using the traditional time constant. Heat conduction modeling within a sheathed thermocouple subject to appropriate boundary conditions, may lead to the derivation of a more descriptive transfer function and explain this anomaly. Evidence is provided here which can be used to assist in relating the level of resolution desired in thermocouple output based on expected fluctuations in input, and how this consideration is related to size and style of thermocouples that are used. But if average temperature is the quantity sought, then thermocouple measurement alone is not likely to provide complete information. Similar statements can be found in most of the literature dealing with thermocouple measurements in fluctuating flames.
